# The Spectrum of Chronic CD8^+^ T-Cell Expansions: Clinical Features in 14 Patients

**DOI:** 10.1371/journal.pone.0091505

**Published:** 2014-03-11

**Authors:** Etienne Ghrenassia, Louise Roulin, Aude Aline-Fardin, Christophe Marzac, Frédéric Féger, Julie Gay, Jérome Pacanowski, Alexandre Hertig, Paul Coppo

**Affiliations:** 1 Service d’Hématologie, Hôpitaux Universitaire de L’Est Parisien, AP-HP, Paris, France; 2 Service d’Anatomie Pathologique, Hôpitaux Universitaire de L’Est Parisien, AP-HP, Paris, France; 3 Laboratoire d’Immuno-Hématologie, Hôpitaux Universitaire de L’Est Parisien, AP-HP, Paris, France; 4 Service de Maladies Infectieuses et Tropicales, Hôpitaux Universitaire de L’Est Parisien, AP-HP, Paris, France; 5 Urgences Néphrologiques et Transplantation Rénale, Hôpitaux Universitaire de L’Est Parisien, AP-HP, Paris, France; 6 Université Pierre et Marie Curie (UPMC), Univ Paris 06, Paris, France; 7 Inserm U1009, Institut Gustave Roussy, Villejuif, France; 8 Centre de Référence des Microangiopathies thrombotiques, Paris, France; Massachusetts General Hospital, United States of America

## Abstract

Chronic CD8^+^ T-cell expansions can result in parotid gland swelling and other organ infiltration in HIV-infected patients, or in persistent cytopenias. We report 14 patients with a CD8^+^ T-cell expansion to better characterize the clinical spectrum of this ill-defined entity. Patients (9 women/5 men) were 65 year-old (range, 25–74). Six patients had ≥1 symptomatic organ infiltration, and 9 had ≥1 cytopenia with a CD8^+^ (>50% of total lymphocyte count) and/or a CD8^+^/CD57^+^ (>30% of total lymphocyte count) T-cell expansion for at least 3 months. One patient had both manifestations. A STAT3 mutation, consistent with the diagnosis of large granular lymphocyte leukemia, was found in 2 patients with cytopenia. Organ infiltration involved lymph nodes, the liver, the colon, the kidneys, the skin and the central nervous system. Three patients had a HIV infection for 8 years (range, 0.5–20 years). Two non-HIV patients with hypogammaglobulinemia had been treated with a B-cell depleting monoclonal antibody (rituximab) for a lymphoma. One patient had a myelodysplastic syndrome with colon infiltration and agranulocytosis. The outcome was favorable with efficient antiretroviral therapy and steroids in HIV-infected patients and intravenous immunoglobulins in 2/3 non-HIV patients. Six patients had an agranulocytosis of favorable outcome with granulocyte-colony stimulating factor only (3 cases), cyclophosphamide, methotrexate and cyclosporine A, or no treatment (1 case each). Three patients had a pure red cell aplasia, of favorable outcome in 2 cases with methotrexate and cyclosporine A; one patient was unresponsive. Chronic CD8^+^ T-cell expansions with organ infiltration in immunocompromised patients may involve other organs than parotid glands; they are non clonal and of favorable outcome after correction of the immune deficiency and/or steroids. In patients with bone marrow infiltration and unexplained cytopenia, CD8^+^ T-cell expansions can be clonal or not; their identification suggests that cytopenias are immune-mediated. Our results extend the clinical spectrum of chronic CD8^+^ T-cell expansions.

## Introduction

Chronic CD8^+^ T-cell expansions, typically composed of large granular lymphocytes (LGL), are reactive (non clonal) or clonal diseases associated with various pathological conditions. Non clonal CD8^+^ T-cell expansions can result in parotid gland swelling and other pseudotumoral organ infiltration in human immunodeficiency virus (HIV)-infected patients, a syndrome termed DILS (diffuse infiltration of CD8^+^ T-cell lymphocytes syndrome). In the setting of allogeneic hematopoietic stem cell transplantation (allo-SCT), chronic CD8^+^ T-cell expansions were identified in long term survivors with chronic graft versus host disease (GVHD) and lymphocytic alveolitis [1]–[5]. Chronic CD8^+^ T-cell expansions were also associated with cytopenia(s) of unexplained origin, such as chronic immunological neutropenia (CIN) and pure red cell aplasia (PRCA), typically in patients with a connective tissue disease [6]–[8]. In these forms, CD8^+^ T-cell expansions may be non clonal, or clonal and then termed LGL leukemia. This latter is characterized by a monoclonal rearrangement of αβ or γδ T-cell receptor (TCR) loci [9]. The distinction between reactive, non clonal CD8^+^ T-cell expansions and LGL leukemia remains challenging but mandatory since their management and their prognosis differ.

Expanded CD8^+^ T lymphocytes, either clonal or not, represent activated cytotoxic T lymphocytes at a terminal stage of their differentiation with evidence of immunological senescence, which have usually lost their cytotoxic properties to become effector memory “regulatory” T lymphocytes [10], [11], [12]. They usually express the CD57 antigen, a surrogate marker of this population, which is also expressed in natural killer cells, and rarely in CD4^+^ T-cells and TCRγδ^+^ T-cells [9]. CD8^+^/CD57^+^ lymphocytes represent 1 to 15% of total lymphocytes in healthy subjects and increase from birth to the elderly [9], [13]. These lymphocytes have oligoclonal restrictions of Vβ and Jβ chains, consistent with an antigen-driven process [14]. In this regard, a CD8^+^/CD57^+^ lymphocytes expansion typically occurs in patients with chronic viral infections and autoimmune diseases, suggesting the chronic stimulation of CD8^+^/CD28^+^/CD57^−^ lymphocytes by exogenous (mostly infection-related), or autologous antigens. In this regard, HIV and cytomegalovirus (CMV) were involved as contributing factors in this process [15], [16]. Paralleling chronic antigen stimulation, these CD8^+^ T-cells acquire a poor capacity to proliferate in standard conditions in relation with the loss of CD28, whereas CD57 antigen becomes expressed at their surface, consistent with an advanced differentiation state and replicative senescence [15], [17]–[20]. The role of these lymphocytes is only partially understood but they could mainly exert immunosuppressive functions which mediators remain to be defined. Alternatively, they were involved in anti-HIV immune response [3], [21], as well as in systemic inflammation with progressive tissue damage [15], [22].

So far, the clinical spectrum of chronic CD8^+^ T-cell expansions remains ill-defined and their management is not consensual, especially in the reactive forms. Here, we performed a retrospective analysis of all CD8^+^ T-cell expansions resulting in tissue infiltration and/or cytopenia(s) over a 6 year period in a single institution. Our aim was to extend the spectrum of clinical features observed in CD8^+^ T-cell expansions and to define relevant indications for which the identification of a CD8^+^ T-cell expansion can be useful in clinical practice. We also provide original insights into the management of this rare condition.

**Table 1:** CD8^+^ T-cell expansion with tissue infiltration in the context of HIV infection.

Patient	Sex/Age	Underlyingconditions	Clinical manifestations	Treatment and outcome	FU(months)
1	M/65	HIV infection	Diarrhea and hepatomegaly withrectal, liver and	ART and symptomatic measures:	50
		Chronic HBV infection	cutaneous CD8^+^ T-cell infiltration	- Ascites evacuation	
		HHV8 infection	NRH – Ascites	- Potassium sparing diuretics	
		Multicentric Castleman’sdisease	Splenomegaly - Lymphnode enlargement	Resolution of clinical manifestations	
				Persistent NRH without complication	
			Pancytopenia	Alive	
2	M/47	HIV infection	Hepatomegaly with liver CD8^+^T-cell infiltration	ART	3
		Chronic HCV infection	Lymph node enlargement	Resolution of clinical manifestations	
		Autoimmune hemolyticanemia	Hyperlymphocytosis	Died of splenic rupture	
			Eosinophilia		
3	M/49	HIV infection	Acute interstitial nephritis	Boluses of methylprednisolone,prednisone 1 mg/kg/day	12
		DILS	Cervicothoracic myelitis andlymphocytic meningitis	ART after 1 month	
			Hyperlymphocytosis	Complete response in 2 weeks.Steroid dependence	
			Hypergammaglobulinemia 76 g/L	Alive	

Clinical features. M: male. HIV: human immunodeficiency virus. HBV: hepatitis B virus. HCV: hepatitis C virus. HHV8: human herpes virus 8. DILS: diffuse infiltration of CD8^+^ T-cell lymphocytes syndrome. NRH: nodular regenerative hyperplasia. ART: antiretroviral therapy. FU: follow-up.

## Materials and Methods

Patients were retrospectively included from May, 2006 to May, 2012. Patients were recruited non-selectively from Hematology, Internal Medicine and Infectious Diseases Departments of Hôpital Saint-Antoine. The study was approved by the institutional review board of Hôpital Saint-Antoine and written informed consent was obtained from patients. Patients had to display a symptomatic organ infiltration by CD8^+^ T-cells, and/or at least one cytopenia, in association with a significant CD8^+^ T-cell expansion in peripheral blood or in bone marrow (>50% of total lymphocyte count and/or a CD8^+^/CD57^+^ T-cell expansion >30% of total lymphocyte count), for at least 3 months. Patients with a peripheral blood or in bone marrow TCRγδ^+^/CD57^+^ T-cell expansion (>5% of total lymphocyte count) were also considered. Patients were classified according to their clinical presentation. Underlying conditions and medications were systematically recorded. Patients presenting an isolated CD8^+^ T-cell expansion and/or a CD8^+^/CD57^+^ T-cell expansion, but neither tissue infiltration nor cytopenia were not retained in the present study. Patient 3 was reported in a previous work [23].

**Table 2:** CD8^+^ T-cell expansion with tissue infiltration in the context of HIV infection.

Patient	HIV load(log/mL)	PBL/mm^3^	LGL onblood smear	CD4^+^PBL/mm^3^ (%)*	CD8^+^PBL/mm3 (%)*	CD4^+^/CD8^+^ratio*	CD8^+^/CD57^+^PBL/mm^3^ (%)*	ANC/ mm^3^	Hb g/dL	Platelets/ mm^3^	Clonality	STATSH2
1	3.1	2100	Yes	378 (18)	1365 (65)	0.28	987 (47)	1240	8.2	48000	Polyclonal	WT
2	4.7	5360	Yes	1130 (21)	2840 (53)	0.45	1290 (24)	4170	11	70000	Oligoclonal	WT
3	4.6	5000	NA	1150 (23)	3388 (68)	0.33	NA	3500	9	276000	Oligoclonal	NA

Biological features.

*performed from PBL immunophenotyping. HIV: human immunodeficiency virus. PBL: peripheral blood lymphocytes. LGL: large granular lymphocytes. ANC: absolute neutrophil count. Hb: hemoglobin. STAT: Signal Transducer and Activator of Transcription. SH2: Src homology-2. WT: wild type. NA: not available.

**Table 3:** CD8^+^ T-cell expansion with tissue infiltration without HIV infection.

Patient	Sex/Age	Underlying conditions	Clinical manifestations	Treatment and outcome	FU(months)
4	F/67	Mantle cell lymphoma	Consciousness disturbances	Interruption of rituximab	44
		Hypogammaglobulinemia(2.2 g/L)	Aseptic meningitis with CD8^+^T-cells on CSF	IVIG	
		Rituximab maintenance	Fever	Resolution of clinicalmanifestations	
			Diarrhea	Alive	
			Hyperlymphocytosis		
5	F/67	Splenic lymphoma withvillous lymphocytes	Wasting	Interruption of rituximaband chemotherapy	41
		Hypogammaglobulinemia(3 g/L)	Diarrhea with CD8^+^ T-cellcolic infiltration	IVIG	
		FCR regimen	Ascites with CD8^+^T-cells – NRH	Resolution of clinicalmanifestations; persistent	
			Pancytopenia	NRH without complication	
				Alive	

Clinical features. F: female. FCR: Fludarabine, cyclophosphamide and rituximab. CSF: cerebrospinal fluid. NRH: nodular regenative hyperplasia. IVIG: intravenous immunoglobulins. FU: follow-up.

**Table 4:** CD8^+^ T-cell expansion with tissue infiltration without HIV infection.

Patient	PBL/mm^3^	LGL onblood smear	CD8^+^ PBL/mm^3^ (%)*	CD4^+^/CD8^+^ratio*	Blood and BMCD8^+^/CD57^+/^mm^3^ (%)*	ANC/mm^3^	Hbg/dL	Platelets/mm^3^	Clonality	STATSH2
4	11410	Yes	9360 (82)	0.12	Blood: 7760 (68)	1710	11.3	168000	Oligoclonal	WT
					BM : 72%					
5	460	Yes	270 (50)	0.64	Blood: 270 (50)	940	7.8	30000	Polyclonal	WT

Biological features.

* performed from PBL immunophenotyping. PBL: peripheral blood lymphocytes. BM: bone marrow. LGL: large granular lymphocytes. ANC: absolute neutrophil count. Hb: hemoglobin. STAT: Signal Transducer and Activator of Transcription. SH2: Src homology-2. WT: wild type.

Patients were evaluated with routine laboratory tests, including blood cell count, bone marrow aspirate and/or bone marrow biopsy. A blood smear with morphologic examination was carried out on all patients. All patients had serologies for acute viral infections (Epstein-Barr virus [EBV], cytomegalovirus [CMV], hepatitis B [HBV] and C [HCV] viruses), as well as for HIV and HTLV-1. CMV reactivation was assessed by genome amplification in blood. In patients with severe neutropenia, anti-surface neutrophil autoantibodies were detected with granulocyte immunofluorescence (GIFT) and with granulocyte agglutination test (GAT) [24]. Detection of anti-neutrophil cytoplasm antibodies (ANCA) and identification of their specificities were performed as reported elsewhere [24].

Lymphocytes immunophenotyping was performed from peripheral blood, ascites or cerebrospinal fluid after mononuclear cell labeling with the following monoclonal antibodies according to standard procedures: CD2, CD3, CD4, CD8, CD14, CD16, CD25, CD45RA, CD45RO, CD56, CD57, TCRαβ, TCRγδ and anti-HLA-DR. Cytogenetic analysis and clonality analysis of blood lymphocytes were performed as previously described [6].

**Table 5:** CD4^+^/CD8^+^ ratio in infiltrated organs.

Patient	Figure	CD4^+^/CD8^+^ ratio
1	1A	0.105
	1C	0.007
	1D	0.54
	1F	0.13
2	1E	0.03
	1H	0*
5	1B	0.09
	1G	0.074

*CD4^+^/CD8^+^ ratio was 0/141.

**Table 6:** Agranulocytosis and PRCA-associated CD8^+^ T-cell expansion.

Patient	Sex/Age	Underlying conditions	Clinical manifestations	Treatment and outcome	FU(months)
6	F/68	Rheumatoid arthritis	Splenomegaly	G-CSF	32
		Sjogren’s syndrome	Agranulocytosis	ANC recovery	
		Type 2 diabetes	Myeloid maturational arrest(Myelocyte-metamyelocyte stage)	1 episode of febrile neutropenia	
			Chronic ulcers, osteitis	Died of pulmonary carcinoma	
7	M/57	Allo-SCT for AML 3 years before	Agranulocytosis	Spontaneous recovery	64
		Hypogammaglobulinemia (2.5 g/L)	Thrombocytopenia	Persistent hyperlymphocytosis	
			Hyperlymphocytosis	Alive	
8	F/66	Rheumatoid arthritis	Agranulocytosis	Cyclophosphamide+rituximab	9
			Myeloid maturational arrest(Myelocyte-metamyelocyte stage)	*E. coli*-bacteriemia, persistantneutropenia	
				Alive	
9	M/44	HCV infection	ANCA-associated agranulocytosis	G-CSF	3
			Acute dermo-hypodermitis	ANC recovery; alive	
			Myeloid maturational arrest(Myelocyte-metamyelocyte stage)		
10	F/25	Cutaneous ichtyosis	Mild neutropenia	Failure of IVIG and steroids	39
			Pure red cell aplasia with BM fibrosis	MTX - CsA – rEPO	
				Hematological recovery; alive	
11	F/56	Urticaria	ANCA-associated agranulocytosis	CsA - G-CSF – Steroids	3
		Raynaud phenomenon	Myeloid maturational arrest(Promyelocyte-myelocyte stage)	ANC recovery - resolution ofarthralgia and urticaria	
		Polyarthritis	Hypergammaglobulinemia	Alive	
12	F/67	Renal transplantation(IgA nephropathy)	Pure red cell aplasia	Rapamycin interruption	12
		Immunosuppressive therapy:rapamycin and tacrolimus	BM infiltration	Failure of rEPO and IVIG	
			Hyperlymphocytosis	Cyclophosphamide-relatedneutropenia	
				Persistent anemia; RBCtransfusions	
				Alive	
13	F/74	Rheumatoid arthritis	Pure red cell aplasia	MTX – rEPO	46
			BM infiltration	Transfusion independence	
			Hyperlymphocytosis	Alive	
			Thrombocytosis		
14	F/70	Myelodysplastic syndrome	Rectal infiltration	MTX and CsA failure	24
			Agranulocytosis	MMF – MTX	
			Anemia	Developed AML	
				Died	

Clinical features. F: female. M: male. Allo-SCT: allogeneic hematopoietic stem cell transplantation. AML: acute myeloblastic leukemia. HCV: hepatitis C virus. ANCA: anti-neutrophil cytoplasm antibodies. BM: bone marrow. ANC: absolute neutrophil count. *E. coli*: *Escherichia coli*. MTX: methotrexate. CsA: cyclosporine A. MMF: mycofenolate mofetil. G-CSF: granulocyte-colony stimulating factor. rEPO: recombinant erythropoietin. IVIG: intravenous immunoglobulins. RBC: red blood cells. FU: follow-up.

**Table 7:** Agranulocytosis and PRCA-associated CD8^+^ T-cell expansion.

Patient	PBL/mm^3^	LGL onblood smear	Blood and BM CD8^+^lymphocytes/mm^3^ (%)*	CD4^+^/CD8^+^ *	Blood and BM CD8^+^/CD57^+/^mm^3^ (%)*	ANC/mm^3^	Hbg/dL	Platelets/mm^3^	Clonality	STAT3SH2
6	1950	Yes	Blood: 819 (42)	0.67	Blood: 555 (32)	200	10.3	188000	Polyclonal	WT
			BM: NA		TCRγδ^+^/CD57^+^T cells: 492 (22)					
7	9330	Yes	Blood: 8676 (93)	0.03	Blood: 8210 (88)	0	11.2	105000	Oligoclonal	WT
			BM: 82%		BM: 67%					
8	6400	Yes	Blood: 3970 (62)	0.5	Blood: 3200 (50)	160	11,8	238000	Clonal	Y640F
			BM: 68%		BM: 54%					
9	2220	No	Blood: 1984 (51)	0.63	Blood: 1400 (36)	0	12.5	288000	Oligoclonal	WT
			BM: 50%		BM: 37%					
10	1460	Yes	Blood: 701 (48)		Blood: 438 (30)	1460	6,2	236000	Clonal	Y640F
			BM: 51%		BM: 40%					
11	2100	Yes	Blood: 731 (34)	0.57	Blood: 301 (14)	100	14.3	168000	Polyclonal	WT
			BM: 38%		BM: 51% including 8%of TCRγδ^+^/CD57^+^ T cells					
12	6400	No	Blood: 3840 (60)	0.5	Blood: 2048 (32)	3300	5.1	221000	NA	NA
			BM: NA							
13	5360	No	Blood: NA		NA	1650	7.1	482000	Oligoclonal	WT
			BM: 86%		BM: 38%					
14	830	No	Blood: 315 (38)	1.4	Blood: 280 (34)	10	8.2	823000	Polyclonal	NA
			BM: 75%							

Biological features.

*performed from PBL immunophenotyping. PBL: peripheral blood lymphocytes. LGL: large granular lymphocytes. BM: bone marrow. TCR: T-cell receptor. ANC: absolute neutrophil count. Hb: hemoglobin. STAT: Signal Transducer and Activator of Transcription. SH2: Src homology-2. WT: wild type. NA: not available.

Clonality of expansion was routinely tested using TCR-γ Polymerase Chain Reaction (PCR) followed by fragment length analysis on 3500 xL Dx Genetic Analyser (Applied Biosystems®) according to Biomed-2 standardization protocol [25]. *STAT3* exon 21 mutations were screened by PCR and high resolution melting curve analysis on LightCycler® 480 instrument (Roche Applied Science) followed by a purification step of amplicons and direct sequencing on 3500 xL Dx Genetic Analyser (primers and conditions available on request).

Median (range) was determined for all continuous variables. Wilcoxon’s test was used to compare continuous variables.

## Results

### General Features

Patients (9 women/5 men) were 65 year-old (range, 25–74). Six patients had ≥1 symptomatic organ infiltration, and 9 had ≥1 cytopenia with a CD8^+^ (>50% of total lymphocyte count) and/or a CD8^+^/CD57^+^ (>30% of total lymphocyte count) T-cell expansion for at least 3 months. One patient had both manifestations. Median peripheral blood lymphocyte count was 4.45 G/L (range, 0.53–11.4 G/L). Seven patients had a peripheral blood lymphocytosis >4 G/L. Median peripheral blood CD8^+^ T-cell count was 1.98 G/L (range, 0.27–9.36 G/L); 7 patients had a CD8^+^ T-cell count >1.5 G/L. CD8^+^/CD57^+^ T-cells involved 35% (range, 14–88%) of total peripheral blood lymphocytes. Bone marrow CD8^+^/CD57^+^ T-cells in patients with cytopenia represented 60% (range, 38–86%) of total bone marrow lymphocytes. Two patients with cytopenia and a CD8^+^/CD57^+^ T-cell expansion in blood or in bone marrow also had a TCRγδ^+^/CD57^+^ polyclonal T-cell expansion, which resulted in a total CD57^+^ T-cell count of >50% in blood or >30% in bone marrow. In all cases, the CD4^+^/CD8^+^ ratio was <2. There was no significant association between peripheral blood lymphocyte count and organ infiltration (median, 5 G/L [range, 0.46–11.4 G/L] in patients with organ infiltration *versus* 2.22 G/L [range, 0.83–9.33] in patients without organ infiltration, p = 0.70). Cytogenetical analysis was performed in four patients and was found normal. T-cells were oligoclonal, polyclonal and clonal in 6 cases, 5 cases and in 2 cases, respectively. The analysis of exon 21 of STAT3 was performed in 12 patients. The Y640F mutation in the SH2 domain of STAT3 was found in 2 patients with clonal TCRγ rearrangement and immunological cytopenias (patients 8 and 10), consistent with the diagnosis of LGL leukemia [26]. No patient had evidence of CMV reactivation.

The median follow-up was 25 months (range, 3–64 months).

### CD8^+^ T-cell Expansion with Tissue Infiltration (Table 1, 2, 3, 4)

**Figure 1:**
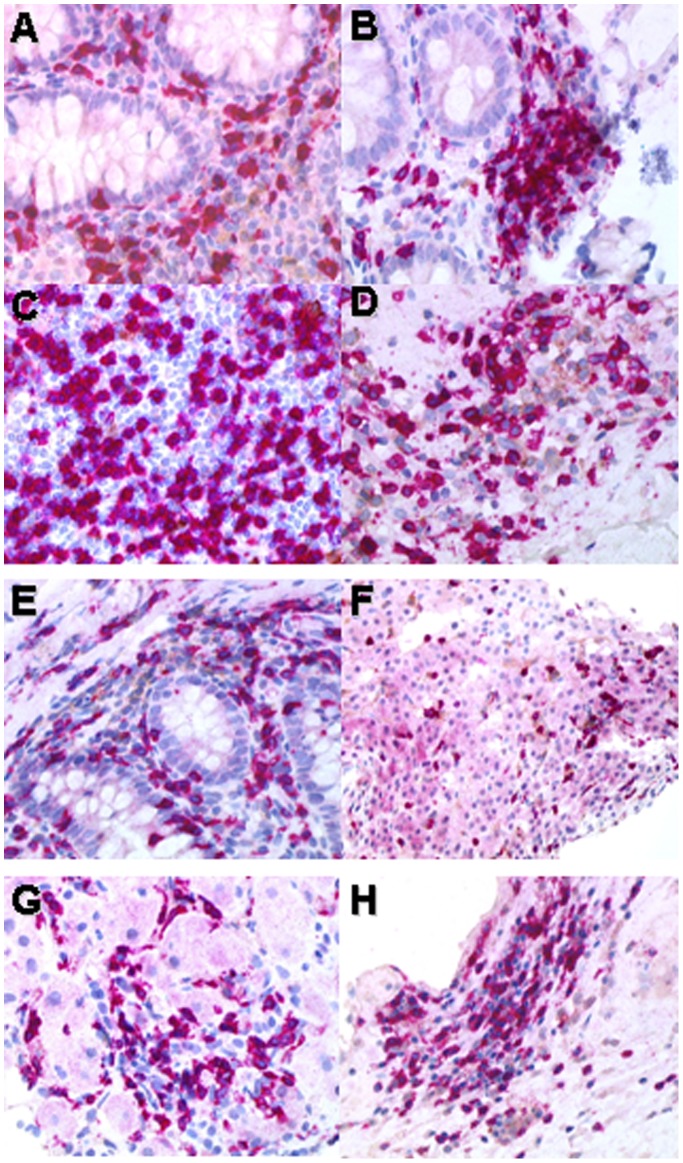
Histopathological findings on patients with CD8^+^ expansion and organ infiltration. CD8 immunostaining on colon (**A**, patient 1; **B**, patient 5), rectum (**C**, patient 1), lymph node (**D**, patient 1, **E**, patient 2), skin (**F**, patient 1) and liver (**G**, patient 5, **H**, patient 2), showing that the lamina propria, the germinal center, the derma and the liver lobules, respectively, are massively infiltrated by CD8^+^ T-cell lymphocytes, whereas CD4^+^ T cell lymphocytes are rare or absent. CD4 was revealed by DAB (brown), and CD8 by Fast Red (magnification x400).

Three patients had a HIV infection for 8 years (5 months-20 years). Two patients had a past history of opportunistic disease: a multicentric Castleman disease (Patient 1), and pulmonary tuberculosis (Patient 3). Median CD4^+^ T cell count at time of CD8^+^ T-cell expansion diagnosis was 0.59 G/L (range, 0.35–1.131 G/L) and HIV RNA viral load was 4.6 Log/mL (range, 3–4.7 log/mL). CD8^+^ T-cell expansion resulted in a peripheral blood lymphocytosis in 2 cases (Patients 2 and 3). Organ infiltration involved the colon and the rectum, the kidney, the cerebrospinal fluid, the skin (1 case each), the liver and lymph nodes (2 cases each) **(** **Figure 1A, 1C–F and 1H** **,** **Table 5** **).** One patient with renal infiltration also had a cervicothoracic myelitis in association with a lymphocytic meningitis, consistent with the diagnosis of DILS [**23**]. Symptoms resolved in all 3 cases with symptomatic measures and efficient ART. The patient with the DILS also required a long term steroid therapy for a steroid-dependent disease [23].

Three patients with a CD8^+^ T-cell expansion-associated tissue infiltration were HIV-negative. Two patients were receiving a maintenance treatment with rituximab (Patient 4) or a fludarabine-based chemotherapy associated with rituximab (Patient 5) for a low grade lymphoma that was in complete remission at time of CD8^+^ T-cell expansion diagnosis. Both patients had a persistent hypogammaglobulinemia. In patient 4, CD8^+^ T-cell expansion occurred 1 year after maintenance treatment with rituximab was started; it was revealed by aseptic CD8^+^ lymphocytic meningitis with fever, diarrhea and lymphocytosis with bone marrow infiltration. No herpes virus infection was evidenced. The patient was treated successfully with intravenous immunoglobulins (0.5 g/kg every 3 weeks for 6 months) and rituximab was suspended. CNS features resolved completely and CD8^+^ T-cell lymphocytes disappeared from CSF. Patient 5 developed an unusually severe pancytopenia during treatment, associated with a persistent diarrhea and a portal hypertension with ascites as a result of a regenerative nodular hyperplasia. CD8^+^ T-cell lymphocytes were found in the ascites liquid, in peripheral blood and in bone marrow. A colon biopsy evidenced a mucosal CD8^+^ T-cell infiltration (**Figure 1B and 1G** **,** **Table 5**). She dramatically improved with intravenous immunoglobulins (0.5 g/kg every 3 weeks for 6 months), with a resolution of diarrhea and a blood cell count recovery. The third patient had a myelodysplastic syndrome with agranulocytosis (detailed below). She had a persistent diarrhea that revealed a CD8^+^ T-cell infiltration in the lamina propria of the colon.

### CD8^+^ T-cell Expansion with CIN and PRCA (Table 6–7)

**Figure 2:**
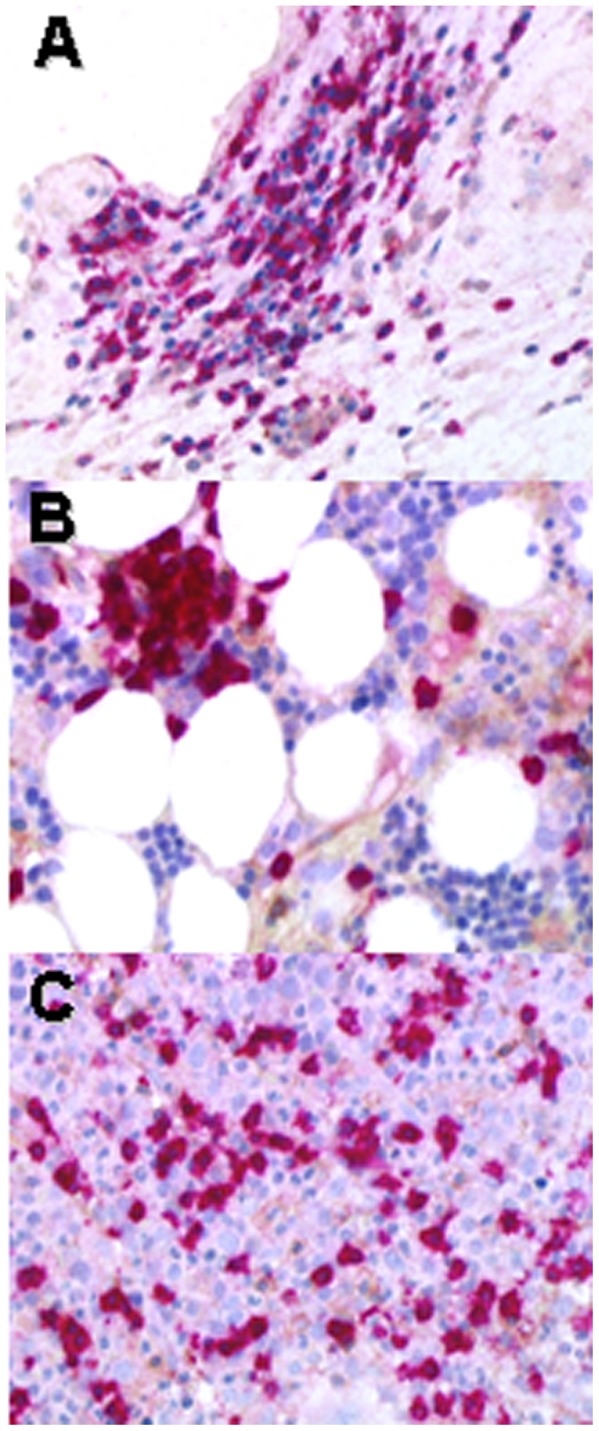
Histopathological findings on patients with CD8^+^ expansion and cytopenia. CD8 immunostaining on rectum (**A**, patient 14) and bone marrow (**B**, patient 14; **C**, patient 10), showing infiltration by numerous CD8^+^T-cell lymphocytes with few CD4^+^ T cells. CD4 was revealed by DAB (brown), and CD8 by Fast Red (magnification x400).

Six patients had an agranulocytosis consistent with a CIN, with no tissue involvement other than blood and/or bone marrow in all but one patient, who also had a rectal infiltration. Patients 7 and 14 also had mild anemia and thrombocytopenia (1 case each). Four patients had an associated disease. Agranulocytosis was revealed by an infectious process in 2 cases: in one case (Patient 9), a dermo-hypodermitis with 2 episodes of severe pneumonia that required mechanical ventilation; in the other case (Patient 14), a febrile neutropenia in a context of myelodysplastic syndrome with a persistent diarrhea that revealed a CD8^+^ T-cell infiltration in the lamina propria of the rectum (**Figure 2A** **,** **Table 8**). In the 4 other cases, agranulocytosis was identified fortuitously while patients were followed-up for an associated condition. Median ANC was 0.06 G/L (range, 0–0.2 G/L). CD8^+^ T-cell expansion resulted in a peripheral blood lymphocytosis in 4 cases (Patients 7, 8, 12, 13). Bone marrow smear disclosed a myeloid maturation arrest in all cases. Bone marrow biopsy was performed in 2 patients and disclosed a CD8^+^ T-cell infiltration (**Figure 2B–C** **,** **Table 8**). Anti-neutrophil antibodies were sought in all patients and were consistently found mildly positive (1+). ANCA were positive in 2/6 patients, consistent with the diagnosis of CIN [24], [27]. Titer was 1/40 and 1/320. They were directed against PR3 in one case, whereas in the other case the specificity could not be identified.

**Table 8:** CD4^+^/CD8^+^ ratio in infiltrated organs in patients with agranulocytosis and PRCA-associated CD8^+^ T-cell expansion.

Patient	Figure	CD4^+^/CD8^+^ ratio
10	2C	0.1
14	2A	0.009
	2B	0.24

Three patients had a PRCA. In 2 cases, it was associated with a connective tissue disease; in the third case, it occurred in a context of renal transplantation performed 27 years before. Median hemoglobin level was 5.7 g/dL (range, 5.1–6.2). PRCA was characterized by a normal growth of erythroblasts *in vitro* with erythropoietin, ruling out an intrinsic defect. No antibodies directed against erythropoietin were found. No patient had thymoma and Parvovirus B19 was negative in all cases.

Five patients had a long standing history of autoimmune disease: a rheumatoid arthritis (Patients 6, 8 and 13), a Raynaud’s phenomenon (Patient 11) and an ichtyosis (Patient 10).

Two patients with agranulocytosis were treated successfully with G-CSF. In a third case, G-CSF allowed a mild increase in neutrophil count. However, a treatment with cyclosporin A and steroids led to durable neutrophil count recovery. One patient was treated successfully with oral cyclophosphamide. One patient with a history of hematopoietic stem cell transplantation recovered neutrophil count spontaneously. The last patient, with a myelodysplastic syndrome, recovered transiently neutrophil count with methotrexate and cyclosporin A before she developed an acute leukemia 2 months later.

Two patients with PRCA were treated successfully with methotrexate, associated with cyclosporine A and recombinant erythropoietin in one case, with a recovery of hemoglobin level. In a third patient, treatment with intravenous immunoglobulins and cyclophosphamide was unsuccessful and the patient remains transfused every 3 weeks.

## Discussion

In our study, patients with a chronic CD8^+^ T-cell expansion presented with two main features. The first was a pseudotumoral organ infiltration by non clonal cells that typically occurred in immunocompromised patients, such as in patients with a HIV infection or in patients receiving an immunosuppressive treatment. The second presentation was an immune cytopenia, typically an agranulocytosis or a PRCA, which may result from either a LGL leukemia [28], or a non clonal expansion in blood and/or bone marrow.

### CD8^+^ T-cell Expansion in Immunocompromised Patients

We identified a CD8^+^ T-cell expansion with organ infiltration in patients with a context of immune deficiency, i.e., a HIV infection and a B cell depletion-based immunosuppressive treatment for a lymphoid malignancy. Studies associated HIV infection with an expansion of CD8^+^ T-cells directed against the virus early in the course of the disease. This expansion is usually transient and followed by a decrease in CD8^+^ T-cell count. The prevalence of a persistent peripheral blood CD8^+^ T-cell lymphocytosis varies from 0.3% to 4.6%, and was associated with a slower progression of the infection and a prolonged survival [3], which is in accordance with our findings since one patient had a HIV infection for more than 20 years. On the opposite, others associated such expansion of activated CD8^+^/HLA DR^+^ T-cells with chronic diseases linked to inflammatory damage and increased morbidity and mortality [15], [16]. In HIV-infected patients, CD8^+^ T-cell lymphocytosis could be observed in the context of DILS [2], [21], [29]. In our patients, parotid gland swelling, a typical feature of DILS, was not present. Rather, organ enlargement involved prominently lymphoid organs with pseudotumoral splenomegaly and hepatomegaly [30], and non-lymphoid organs such as the digestive tract, the skin, the CNS and kidneys. Therefore, our findings widen the spectrum of CD8^+^ T-cell organ infiltration in HIV-infected patients. Importantly, the identification of a non clonal CD8^+^ T-cell expansion, and more particularly if a majority of CD8^+^ T-cells express the CD57 antigen, may help to rule out the diagnosis of lymphoid malignancy in these patients and avoid performing a splenectomy or other invasive diagnostic explorations. In this context, the diagnostic value of the fluorodeoxyglucose positron-emitting tomography deserves evaluation.

We report a CD8^+^ T-cell expansion with organ infiltration in 2 patients treated with immunosuppressive drugs. Interestingly, both patients shared similar features: an advanced age (67 year-old both), organ infiltration (i.e., lymphocytic meningitis in 1 case and a chronic diarrhea with a digestive tract infiltration and ascitis with CD8^+^ T-cells in the other), a severe hypogammaglobulinemia, and an immunosuppressive chemotherapy including multiple infusions of rituximab. These features strongly suggest a role for infectious agents occurring within an underlying immune deficiency (an advanced age, a profound hypogammaglobulinemia and a prolonged treatment with immunosuppressive drugs), and a role for an antigen-driven response in the occurrence of CD8^+^ T-cell expansion. Consistent with this hypothesis, the outcome was remarkably favorable in both cases with rituximab interruption and intravenous immunoglobulins. Moreover, this clinical presentation was reminiscent of those observed in DILS in the era of efficient ART, with a predominance of extraglandular manifestations [29]. Cases of CD8^+^ T-cell expansions in the setting of hypogammaglobulinemia and rituximab treatment still remain poorly reported in literature [31]–[34]; therefore, clinicians should be aware about this diagnosis.

The mechanisms leading to non clonal, reactive CD8^+^ T-cell expansions in these settings are not fully understood. Animal models strongly argue for an important role of an underlying immune deficiency, which may result in a dysfunction in the usual contraction process that follows the expansion of activated CD8^+^ T-lymphocytes after an antigenic challenge, either autologous in a context of autoimmune disease or exogenous in a context of chronic infectious disease. Moreover, this dysfunctional process may alter the repartition of immunodominant T-cell populations, accounting for the predominance of expanded clones in these patients [35], [36]. In the specific case of DILS, Itescu *et al*. suggested that some immunogenetically distinct individuals were responding to HIV infection by developing ‘reactive’ circulating CD8^+^ lymphocytosis, and that lymphocytes were sequestered into the different organs by interaction with accessory molecules. However, it cannot be excluded that the expansion of CD8^+^ T-cells could also be due to other viral coinfectants such as EBV or HTLV-1 [21].

### CIN and PRCA-associated CD8^+^ T-cell Expansion

In adults, CIN and autoimmune PRCA are typically mediated by autoimmune cytotoxic T-cells through the release of inhibitory molecules [7], [8]. They can be variably associated with conditions such as rheumatoid arthritis and Felty’s syndrome, Sjogren’s syndrome and systemic lupus erythematosus, as well as chronic GVHD, lymphoid malignancies and primary immune deficiencies ([6]–[8]; present study). These conditions may occur in the context of LGL leukemia; alternatively, they may represent exclusion diagnoses made after the other causes of neutropenia and PRCA have been excluded. In this context, the presence of reactive, oligoclonal CD8^+^ T-cells with features of LGL (which was termed the syndrome of T8 hyperlymphocytosis by previous authors, [5], [37]) or more rarely TCRγδ^+^/CD8^+^ T-cells ([38], present study) can be of diagnostic help in patients explored for a chronic neutropenia or a PRCA of unexplained origin. Noteworthy, CD8^+^ T-cells expansion may not be evident from morphological examination of the bone marrow aspirate or biopsy, from flow cytometric analysis of the aspirate, or from the peripheral blood lymphocyte count. Therefore, immunohistochemical analysis should be performed routinely in these rare conditions [39]. Interestingly, it is not excluded that oligoclonal CD8^+^ T-cell expansions and LGL leukemia may represent a continuous spectrum in which oligoclonal, chronically stimulated CD8^+^ lymphocytes may finally acquire oncogenic events until an overt LGL leukemia develops. Recently, the pathophysiological mechanism of LGL leukemia was outstandingly improved with the recent identification of acquired mutations of the SH2 domain of *STAT3* and *STAT5b* in 40% and 2% of cases, respectively [26], [40], and the search for these mutations may help to distinguish LGL leukemia from reactive CD8^+^ T-cell expansions.

A pathogenic CD8^+^ T-cell expansion was associated with anemia and/or neutropenia in allo-SCT ([4]; present study) through a HLA class II-restricted inhibition of bone marrow erythroblastic and/or granulocytic progenitors, respectively [41]. In this context, CD8^+^ T-cell expansions were associated with a prolonged relapse free survival, suggesting that CD8^+^ T-cells recognize epitopes in malignant cells [4]. Therefore, their identification in this context may be of prognostic value. In this context, CD8^+^ T-cells were reported to have a suppressive effect on hematopoiesis, which could be the consequence of an allogeneic reaction [41]. Similarly, self-reactive CD8^+^ T-cells may recognize autologous antigens, and could result in CIN [37].

The identification of a CD8^+^ T-cell expansion was also observed in myeloid malignancies such as acute leukemias and myelodysplastic syndromes. In these latter, CD8^+^ T-cells were found to recognize self-antigens in up to 80% of cases, which may account for the severe cytopenias we observed in one patient of the present series. Interestingly, somatic mutations of the SH2 domain of *STAT3* were identified in the lymphoid compartment in patients with myelodysplastic syndrome and profound neutropenia, consistent with the presence of subclinical T-cell clones which may be involved in the development of hypocellularity. In these patients, CD8^+^ T-cell expansions may be associated with a better response to immunosuppressive treatments [42].

In conclusion, CD8^+^ T-cell expansions can result in the infiltration of various organs, including blood and bone marrow. They typically involve the senescent CD8^+^/CD57^+^ T-cells subpopulation and should therefore be considered as a specific syndrome. The search for a CD8^+^ T-cell expansion should be performed in immunocompromised patients who present with a symptomatic organ infiltration, including blood and bone marrow when patients present with unexplained cytopenia(s). The study of the underlying pathological context, the cytogenetical and TCR-γ clonality analyses and the search of mutations in the SH2 domain of *STAT3* and *STAT5b* should help to distinguish LGL leukemia or other malignant lymphomas from non clonal CD8^+^ T-cell expansions. In patients with a smoldering organ enlargement, the identification of a CD8^+^ T-cell expansion can help to rule out a lymphoid malignancy and to avoid invasive explorations, whereas in patients with unexplained cytopenia, such a population suggests an autoimmune process. The identification of an organ enlargement attributed to a CD8^+^ T-cell expansion may also have therapeutical consequences. This condition may therefore represent an indication to reevaluate or initiate an efficient ART in HIV infected patients, in association with steroids in selected cases; in immunocompromised non HIV-infected patients, intravenous immunoglobulins (and, more generally, the correction of the immune deficiency) may help to resolve clinical manifestations. In patients with a symptomatic cytopenia, the identification of a CD8^+^ T-cell expansion may require hematopoietic growth factors and/or immunomodulatory drugs. Though the monocentric design of our study represents a limitation, it allowed our patients to be explored homogeneously. Another limitation of this study is the small number of reported patients. Consequently, our preliminary data require future prospective studies with a large screening of CD8^+^/±CD57^+^ T-cells in selected cohorts of patients with HIV infection and cytopenia to confirm their diagnostic and prognostic value, which could be incorporated in diagnostic and predictive scores.
